# δ-opioid receptor activation protects against Parkinson’s disease-related mitochondrial dysfunction by enhancing PINK1/Parkin-dependent mitophagy

**DOI:** 10.18632/aging.103970

**Published:** 2020-11-10

**Authors:** Yuan Xu, Feng Zhi, Jiahao Mao, Ya Peng, Naiyuan Shao, Gianfranco Balboni, Yilin Yang, Ying Xia

**Affiliations:** 1 Department of Neurosurgery, The First People’s Hospital of Changzhou, Changzhou, Jiangsu, China; 2 Modern Medical Research Center, The Third Affiliated Hospital of Soochow University, Changzhou, Jiangsu, China; 3 Department of Life and Environment Sciences, University of Cagliari, Cagliari, Italy; 4 Shanghai Key Laboratory of Acupuncture Mechanism and Acupoint Function, Department of Aeronautics and Astronautics, Fudan University, Shanghai, China

**Keywords:** δ-Opioid receptor, mitochondria, PINK1, Parkin

## Abstract

Our previous studies have shown that the δ-opioid receptor (DOR) is an important neuroprotector via the regulation of PTEN-induced kinase 1 (PINK1), a mitochondria-related molecule, under hypoxic and MPP^+^ insults. Since mitochondrial dysfunctions are observed in both hypoxia and MPP^+^ insults, this study further investigated whether DOR is cytoprotective against these insults by targeting mitochondria. Through comparing DOR-induced responses to hypoxia versus MPP^+^-induced parkinsonian insult in PC12 cells, we found that both hypoxia and MPP^+^ caused a collapse of mitochondrial membrane potential and severe mitochondrial dysfunction. In sharp contrast to its inappreciable effect on mitochondria in hypoxic conditions, DOR activation with UFP-512, a specific agonist, significantly attenuated the MPP^+^-induced mitochondrial injury. Mechanistically, DOR activation effectively upregulated PINK1 expression and promoted Parkin’s mitochondrial translocation and modification, thus enhancing the PINK1-Parkin mediated mitophagy. Either PINK1 knockdown or DOR knockdown largely interfered with the DOR-mediated mitoprotection in MPP^+^ conditions. Moreover, there was a major difference between hypoxia versus MPP^+^ in terms of the regulation of mitophagy with hypoxia-induced mitophagy being independent from DOR-PINK1 signaling. Taken together, our novel data suggest that DOR activation is neuroprotective against parkinsonian injury by specifically promoting mitophagy in a PINK1-dependent pathway and thus attenuating mitochondrial damage.

## INTRODUCTION

Mitophagy is an intracellular quality control mechanism that selectively degrades damaged mitochondria and is required for neuronal function and survival [1, 2]. The pathway involving PTEN-induced kinase 1 (PINK1) and Parkin (E3 ubiquitin ligase) is the best understood regulator of mitophagy [3]. In general, mitochondrial membrane potential depolarization or the accumulation of misfolded mitochondrial proteins inhibits presenilin-associated rhomboid-like protein (PARL)-induced PINK1 cleavage, thereby stabilizing PINK1 in the outer mitochondrial membrane (OMM) [4]. Then, Parkin is recruited to the OMM and activated by PINK1 through phosphorylation of Serine 65 in its ubiquitin-like (UBL) domain [5]. Finally, the activated Parkin initiates mitophagy by ubiquitinating several proteins in the OMM [4, 6, 7].

The mitochondria are major sites of cellular energy production, cytosolic calcium buffering and biosynthesis metabolism, which is closely linked with the life and death of eukaryotic cells. Therefore, the failure of the mitochondrial quality control (mitoQC) machinery results in mitochondrial dysfunction and thus is involved in the pathogenesis of several human diseases [5]. One of the typical examples is the autosomal recessive form of Parkinson’s disease (PD). Its pathogenesis is closely related to the mutations of the genes encoding PINK1 and Parkin, two major players in mitochondrial quality control [8]. Several studies have shown that *PINK1* or *Parkin* knockout animal models exhibit significant mitochondrial dysfunction and demonstrate typical PD-like symptoms, including loss of dopaminergic neurons and motor deficits [9–11]. Sun et al used transgenic mice expressing the mitochondrial-targeted pH-dependent fluorescent Keima protein (mt-Keima mice) and observed 70% reduction in mitophagy in the older mice in the dentate gyrus region of the brain, which is critical for memory and learning [12]. Mitochondrial complex I deficiency and mitochondrial DNA aberrations have also been reported in the brain, skeletal muscle tissues, and platelets of individuals with PD [13, 14]. Therefore, mitochondrial defects are implicated in the process of senescence and neurodegeneration. Besides, mitochondrial dysfunction also renders cerebral neurons more susceptible to hypoxic injury [15]. The cerebral neurons are enriched in mitochondria that provide energy required through oxidative phosphorylation (OXPHOS) for the neuronal activity [16]. Functional alterations in the mitochondrial function in response to insufficient oxygen and blood supply to the brain cause irreversible neuronal injury and brain damage [17]. In such a scenario, ATP generation is reduced and deleterious reactive oxygen species (ROS) such as superoxide radicals are generated in excessive amounts [17, 18]. Subsequently, hypoxic and/or ischemic stress causes significant neuronal damage because of progressive loss of mitochondrial function.

Since mitoQC plays a key role in protecting the neurons, modulation of mitophagy has been investigated as a potential strategy for the prevention and treatment of PD and ischemic/hypoxic stroke. The δ-opioid receptor (DOR) is highly expressed in the brain striatum and acts as a modulator of dopamine, GABA, and glutamate neurotransmission [19]. The levels of these opioid peptides, which are released as chemical messengers or neurotransmitters by the striatal neurons, are significantly altered in the brains of patients with PD [20]. Several studies supported the close linkage between DOR and PD pathogenesis [21, 22]. More recently, our work have shown that DOR protects neurons against hypoxia and 1-methyl-4-phenyl-pyridimium (MPP^+^) by up-regulating PINK1 [23–25]. However, it is still unclear whether DOR is involved in the PINK1-Parkin mediated mitoQC, and protects the neurons through the same mechanisms in the parkinsonian insults vs. hypoxia stress. Since highly differentiated PC12 cell line is one of the most commonly used neuron-like cell lines and widely used for the establishment of *in-vitro* model of nerve diseases [26–29], and we used PC12 cells to induce PD injury and hypoxic/ischemic injury in our series studies and gained reliable results [24, 25, 30], we continued to investigated the possible mechanisms through which DOR protects against PD- and hypoxia-related neuronal injury using PC12 cells as an *in vitro* cellular model in this work. Our new results have well demonstrated a novel role of DOR against parkinsonian injury by specifically promoting mitophagy in a PINK1-dependent pathway, which may provide a new target for the prevention and/or treatment of PD.

## RESULTS

### DOR activation attenuated MPP^+^-induced mitochondrial potential depolarization and functional damage, but had a less effect on those of hypoxia-exposed cells

**Figure 1:**
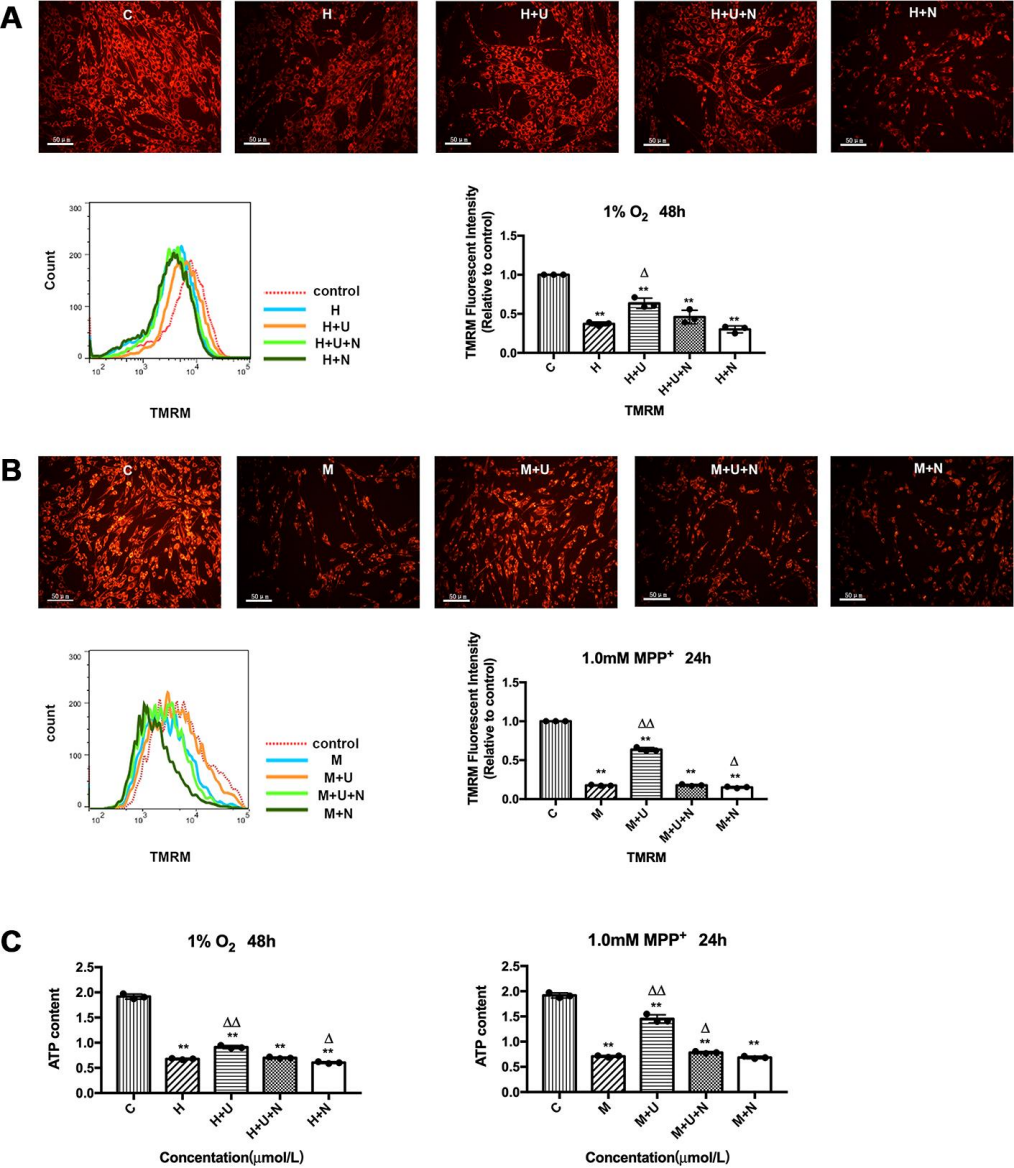
**DOR activation attenuated hypoxic and/or MPP^+^ insults induced mitochondrial membrane potential depolarization and mitochondrial dysfunction.** (**A**) PC12 cells were exposed to hypoxia at 1% O_2_ for 48 hrs, the mitochondrial membrane potential was measured using TMRM reagent. C: normoxic control. H: hypoxia. H+U: DOR was activated using UFP-512 in hypoxic conditions. H+U+N: PC12 cells were treated with UFP-512 plus naltrindole at the same time in hypoxic conditions. H+N: PC12 cells were treated with DOR antagonist naltrindole alone in hypoxic condition. N=3 in each group. ^**^p<0.01 vs. C; ^Δ^*p*<0.05 vs. H; Note that hypoxia significantly decreased the red fluorescent intensity compared to the control, suggesting the collapse of mitochondrial membrane potential. The application of DOR agonist UFP-512 attenuated these changes and the flow cytometer results were consistent with the fluorescence microscope observation. (**B**) PC12 cells were exposed to 1.0 mM MPP^+^ for 24 hrs. C: control. M: MPP^+^. M+U: DOR was activated using UFP-512 and exposed to MPP^+^. M+U+N: PC12 cells were treated with UFP-512 plus naltrindole and exposed to MPP^+^. M+N: PC12 cells were exposed to naltrindole along with MPP^+^. N=3 in each group. ^**^p<0.01 vs. C; ^Δ^*p*<0.05, ^ΔΔ^*p*<0.01 vs. M. Note that MPP^+^ insults also caused a depolarization of mitochondrial membrane potential, while activating DOR using UFP-512 significantly reversed these destructive changes induced by MPP^+^ insults. In contrast, applying DOR antagonist naltrindole alone further aggravated the collapse of mitochondrial membrane potential under MPP^+^ insults. The results measured by flow cytometer were consistent with the florescence observation. (**C**) PC12 cells were exposed to 1% O_2_ for 48 hrs or 1.0mM MPP^+^ for 24 hrs. N=3 in each group. ^**^p<0.01 vs. C; ^Δ^*p*<0.05, ^ΔΔ^*p*<0.01 vs. H or M. Note that hypoxia and/or MPP^+^ caused a significant decrease in ATP generation, while DOR activation restored the capacity of mitochondria in ATP production.

To determine whether the DOR-mediated neuroprotection is realized by targeting mitochondria, we firstly detected the alternations in mitochondrial membrane potential using TMRM reagent. As Figure 1A and 1B depicted, mitochondrial health maintained a difference in electrical potential between the interior and exterior of the organelle, which allowed the TMRM dye to enter the mitochondrial matrix and be aggregated, thus fluorescing red. However, after the cells were exposed to hypoxia at 1% of O_2_ for 48 hrs or 1.0mM MPP^+^ for 24 hrs, TMRM accumulation decreased and the fluorescence signal diminished, suggesting a loss of mitochondrial membrane potential (Figure 1A, 1B). The cells incubated with specific DOR agonist UFP-512 showed a brighter fluorescence compared to the cells exposed to hypoxia and/or MPP^+^ alone (Figure 1A, 1B). This was especially true in MPP^+^ condition. As shown in Figure 1B, the application of UFP-512 greatly increased the TMRM fluorescent intensity, whereas the addition of DOR antagonist, naltrindole (1 μM) completely reversed the effect induced by DOR activation. We also used naltrindole alone to treat the cells exposed to either hypoxia or MPP^+^, which significantly aggravated mitochondrial membrane potential depolarization under MPP^+^ insults, but showed an inappreciable effect on the cells exposed to hypoxia (Figure 1A, 1B). The mitochondrial membrane potential was also evaluated using JC-1 Mitochondrial Membrane Potential Assay Kit by measuring the ratio of red/green fluorescence (Supplementary Figure 1A–1C). The data were consistent with the conclusions obtained using TMRM.

Mitochondria are considered to be the “power factory” of eukaryote. We therefore evaluated the mitochondrial function by measuring the alternations in ATP generation under hypoxic and MPP^+^ stress. Our results showed that hypoxia and MPP^+^ significantly reduced the ATP content by 64.7% and 63.0% respectively, compared to the control (Figure 1C). UFP-512 led to a dramatic increase in the ATP generation (+104.7% in MPP^+^), and a slightly increase in the ATP generation (+34.9% in hypoxia), restoring the capacity of mitochondria in PC12 cells (Figure 1C). Naltrindole, added along with UFP-512, abolished the beneficial effects of UFP-512 on ATP production recovery and even further impaired mitochondrial function when naltrindole was applied to the cells alone (Figure 1C).

### DOR protected PC12 cells from apoptosis through differential regulation of ROS under hypoxic vs. MPP^+^ stress.

Since ROS is mainly originated from mitochondrial respiration, mitochondrial dysfunction has long been thought as a trigger for overproduced and unregulated ROS production [31]. We therefore evaluated the effects of DOR activation on ROS regulation by using DCFH-DA reagent, a probe for total ROS, and MitoSOX, an indicator that specifically targets the mitochondrial superoxide, respectively. As shown in Supplementary Figure 2A and 2B, both hypoxic and MPP^+^ insults induced a remarkable increase in ROS. When the PC12 cells were exposed to 1% O_2_ of hypoxia for 48 hrs, ROS was up-regulated by 87.6 %. Activating DOR using UFP-512 slightly reduced ROS release by 4.0% (Supplementary Figure 2B). In contrast, the cells treated with 1.0mM MPP^+^ for 24 hrs showed a 40.7% increase in the ROS fluorescent intensity, which was significantly attenuated by 23.8% after the application of UFP-512. The effect induced by DOR activation was fully reversed by the naltrindole treatment under both hypoxia and MPP^+^ conditions (Supplementary Figure 2A, 2B).

**Figure 2:**
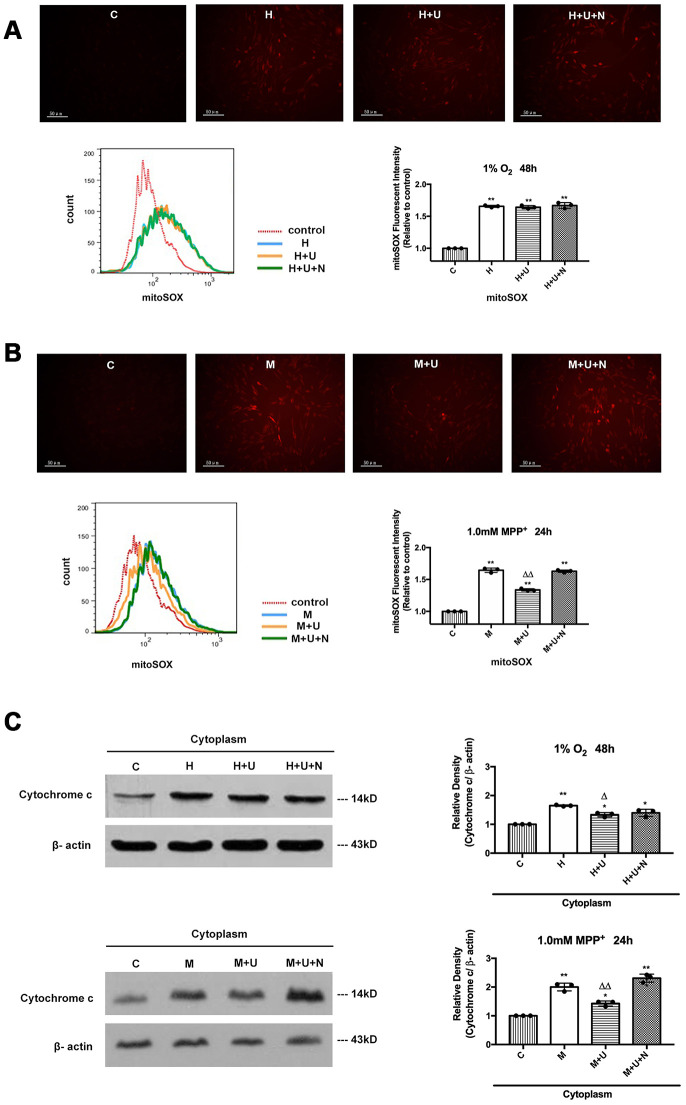
**DOR activation strongly protected PC12 cells from the oxidative injury induced by MPP^+^, and inhibited the cytochrome c release with a mild effect on the cells exposed to hypoxia.** (**A**) PC12 cells were exposed to 1% O_2_ for 48 hrs. The mitochondrial superoxide was detected by MitoSOX Red Mitochondrial Superoxide Indicator. C: normoxic control. H: hypoxia. H+U: DOR was activated using UFP-512 in hypoxic conditions. H+U+N: PC12 cells were treated with UFP-512 plus naltrindole at the same time in hypoxic conditions. N=3 for each group. ^**^p<0.01 vs. C. Note that hypoxia significantly increased the mitochondrial superoxide appearing as a sharp increase in red fluorescent intensity, incubation the cells with DOR specific agonist UFP-512 showed no appreciable difference. (**B**) PC12 cells were treated with 1.0mM MPP^+^ for 24hrs. C: control. M: MPP^+^. M+U: DOR was activated using UFP-512 and exposed to MPP^+^. M+U+N: PC12 cells were treated with UFP-512 plus naltrindole and exposed to MPP^+^. N=3 for each group. ^**^p<0.01 vs. C; ^ΔΔ^*p*<0.01 vs. M. Note that MPP^+^ up-regulated mitochondrial superoxide in PC12 cells. DOR activation significantly attenuated the superoxide generation with a remarkable decrease in red fluorescent density under MPP^+^ insults, while the addition of naltrindole plus UFP-512 reversed the effects induced by DOR activation. (**C**) PC12 cells were exposed to 1% O_2_ for 48 hrs or treated with 1.0mM MPP^+^ for 24 hrs, the protein from cytosol were extracted and analyzed by Western blot. N=3 for each group. ^*^p<0.05, ^**^p<0.01 vs. C; ^Δ^*p*<0.05 vs. H; ^ΔΔ^*p*<0.01 vs. M. Note that hypoxia and MPP^+^ caused a rise of cytochrome c in the cytoplasm, whereas DOR activation remarkably inhibited the release of cytochrome c from the mitochondria to the cytoplasm against MPP^+^ insults with a mild effect on the cells exposed to hypoxia.

Then, we investigated the effects of DOR on mitochondrial superoxide by using MitoSOX indicator. We found that hypoxia and MPP^+^ caused a remarkable increase in mitochondrial superoxide with a strong red fluorescence, while UFP-512 incubation significantly decreased the red fluorescence intensity in the condition of MPP^+^, but showed an inappreciable effect on the cells exposed to 1% O_2_ of hypoxia (Figure 2A, 2B). The addition of DOR antagonist naltrindole completely reversed these beneficial changes under MPP^+^ insults.

Furthermore, we examined the expression level of pro-apoptotic protein, cytochrome c, which is released from mitochondria to cytosol when the cell apoptosis pathway is activated [32]. Hypoxia and MPP^+^ caused a significant rise of cytochrome c in the cytoplasm, along with the increase in ROS, whereas DOR activation largely blocked the release of cytochrome c from mitochondria to cytoplasm in MPP^+^ condition, but induced a less change in hypoxic condition (Figure 2C).

### DOR knockdown or PINK1 knockdown aggravated mitochondrial damage and deprived the DOR-mediated mitoprotection in both hypoxic and MPP^+^ insults

**Figure 3:**
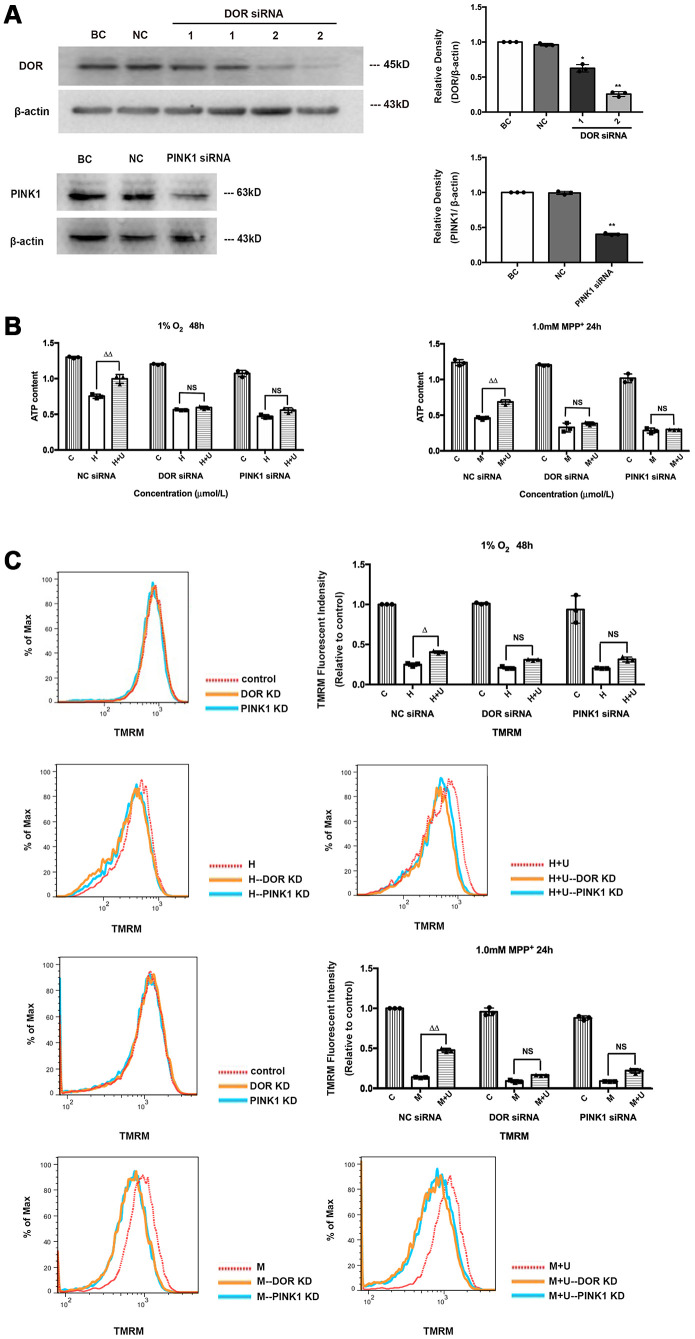
**Beneficial effects of DOR activation on mitochondria under hypoxic and/or MPP^+^ insults were partially blocked by PINK1 knockdown or DOR knockdown.** (**A**) BC: blank control. PC12 cells were merely transfected with lipofectamine 2000. NC: negative control. PC12 cells were transfected with negative control siRNA. DOR siRNA: PC12 cells were tranfected with two kinds of DOR siRNA 1 and 2. PINK1 siRNA: PC12 cells were transfected with PINK1 siRNA. N=3 for each group. ^*^p<0.05, ^**^p<0.01 vs. BC. Note that the expression intensity of DOR was significantly interfered by DOR siRNA 2, and PINK1 expression was significantly reduced by PINK1 siRNA transfection. (**B**) PC12 cells were exposed to 1% O_2_ for 48 hrs or 1.0mM MPP^+^ for 24 hrs. C: control. H: hypoxia. H+U: DOR was activated using UFP-512 in hypoxic conditions. M: MPP^+^. M+U: DOR was activated using UFP-512 and exposed to MPP^+^. N=3 in each group. ^ΔΔ^*p*<0.01 vs. H or M; NS: not significant. Note that DOR knockdown or PINK1 knockdown caused a significant decrease in ATP production both under the conditions of hypoxia and/or MPP^+^. Treat cells with DOR agonist failed to restore the capacity of mitochondria in ATP generation after cells were transfected with DOR siRNA or PINK1 siRNA. (**C**) PC12 cells were exposed to 1%O_2_ for 48 hrs or 1.0 mM MPP^+^ for 24 hrs, the mitochondrial membrane potential was measured using TMRM. N=3 in each group. NS: not significant; ^Δ^*p*<0.05 vs. H; ^ΔΔ^*p*<0.01 vs. M. Note that the knockdown of DOR or PINK1 significantly blocked the effects of DOR activation on attenuating mitochondrial membrane potential collapse both under hypoxic and/or MPP^+^ insults.

In previous studies, we have demonstrated that the DOR-mediated neuroprotection against hypoxic and MPP^+^ insults was majorly dependent on PINK1 signaling [25]. Assuming that the DOR-PINK1 axis is a critical component of the mechanisms for mitoprotection against parkinsonian injury, we first asked if the knockdown of DOR or PINK1 affects the DOR-mediated mitoprotection. The PC12 cells were transfected with DOR siRNA, PINK1 siRNA, or the negative control siRNA. Compared to the cells transfected with negative control siRNA, the cells transfected with PINK1 siRNA showed an at least 50% reduction in PINK1 proteins, and the DOR expression in the cells transfected with DOR siRNA was also reduced by almost 70% (DOR siRNA 2) (Figure 3A).

Then, we analyzed the alternations in mitochondrial membrane potential and ATP production following hypoxic and MPP^+^ insults before and after DOR/ PINK1 knockdown. The results showed that DOR knockdown or PINK1 knockdown did not seriously alter mitochondrial function and structure in the control group, but largely accelerated the collapse of mitochondrial potential, and impaired mitochondrial function under both hypoxia and MPP^+^ conditions (Figure 3B, 3C). Compared to the cells transfected with negative control siRNA, the cells transfected with DOR siRNA and the cells transfected with PINK1 siRNA also caused a major loss in the DOR -mediated mitoprotection. As mentioned above, DOR activation significantly attenuated the collapse of mitochondrial membrane potential, restored mitochondrial function against hypoxic and MPP^+^ insults, which is especially true under MPP^+^ conditions. However, the beneficial effects of DOR agonist on mitochondria were remarkably deprived after the knocking down of DOR or PINK1, appearing as negligible differences in mitochondrial membrane potential and ATP production between “H” and “H+U” groups or between “M” and “M+U” groups (Figure 3B, 3C).

**Figure 4:**
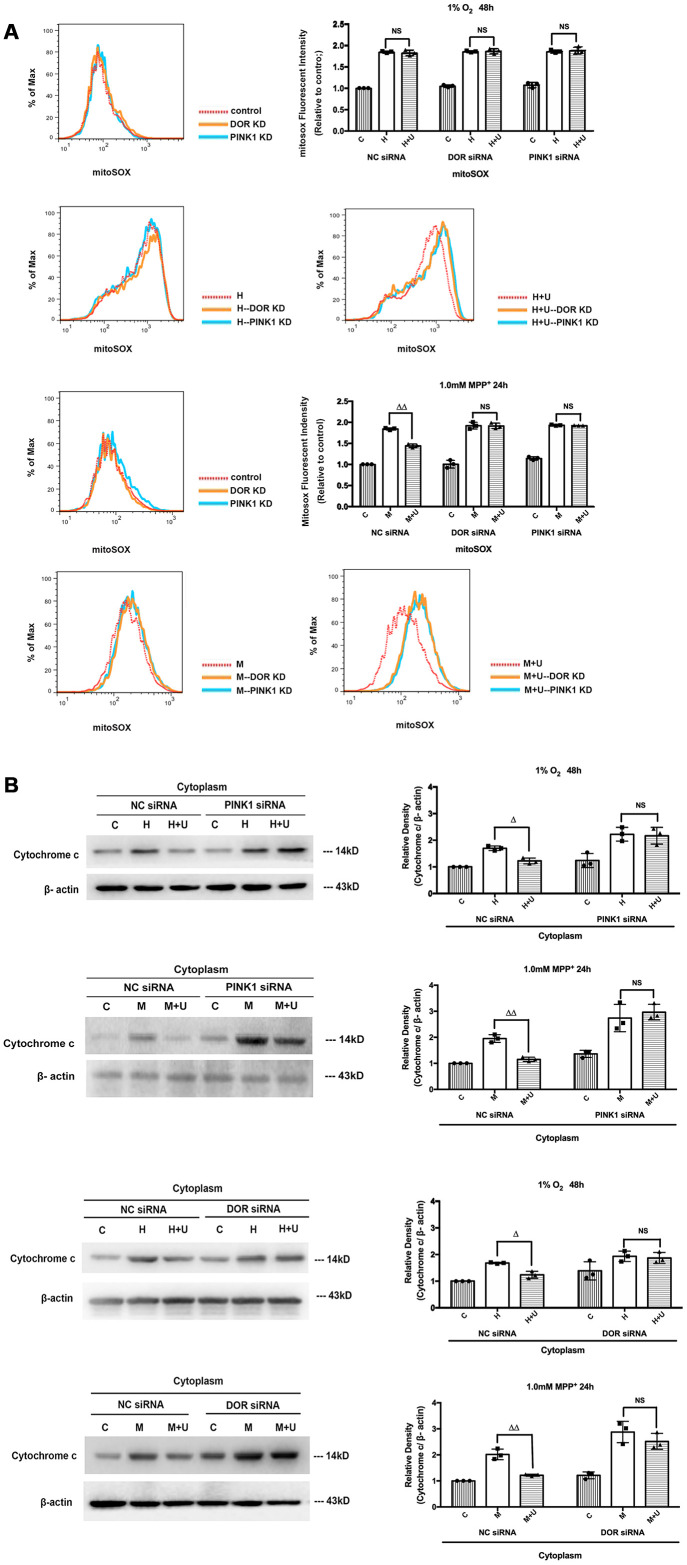
**Knockdown of DOR or PINK1 interfered with DOR-mediated cytoprotection against hypoxia and/or MPP^+^.** (**A**) The PC12 cells transfected with DOR siRNA or PINK1 siRNA together with the cells transfected with NC siRNA were exposed to hypoxia at 1% O_2_ for 48 hrs or 1.0mM MPP^+^ for 24 hrs, and then the superoxide fluorescence was detected using MitoSOX Red Mitochondrial Superoxide Indicator. C: control. H: hypoxia. H+U: DOR activation with UFP-512 in hypoxic condition. M: MPP^+^. M+U: DOR was activated using UFP-512 and then exposed to MPP^+^. N=3 in each group. NS: not significant; ^ΔΔ^*p*<0.01 vs. M within the same group. Note that the statistical data analyzed by flow cytometer showed that DOR knockdown or PINK1 knockdown greatly interfered with the effects of DOR activation on mitochondrial superoxide especially under MPP^+^ conditions. (**B**) PC12 cells transfected with DOR siRNA or PINK1 siRNA together with the control were exposed to hypoxia at 1% O_2_ for 48 hrs or 1.0mM MPP^+^ for 24 hrs, cytochrome c in the cytosol were extracted and measured using Western blot. N=3 in each group. NS: not significant; ^Δ^*p*<0.05, ^ΔΔ^*p*<0.01 vs. H or M within the same group. Note that the knockdown of PINK1 or DOR significantly increased the release of cytochrome c from mitochondria to cytosol under hypoxic and/or MPP^+^ insults. The effects of DOR activation on cytochrome c were largely deprived by PINK1 knockdown or DOR knockdown.

Subsequently, we investigated whether DOR knockdown or PINK1 knockdown further interfered with the effects of DOR on ROS. Our data suggested that PINK1 knockdown accelerated the mitochondrial based cell injuries under hypoxic and MPP^+^ insults with a significant increase in the ROS level (Supplementary Figure 3A, S3B). In sharp contrast to the cells transfected with negative control siRNA, the cells transfected with PINK1 siRNA did not exhibit any beneficial regulation in ROS release after the administration of UFP-512 (Supplementary Figure 3A, 3B). Furthermore, we examined the alternations in superoxide in the mitochondria. As Figure 4A depicted, the mitochondrial superoxide was largely eliminated by DOR agonist UFP-512 under MPP^+^, but not under hypoxia in the negative control siRNA transfection group. DOR knockdown or PINK1 knockdown largely interfered with the elimination of mitochondrial superoxide by DOR activation with a significant increase in fluorescence intensity under MPP^+^ (Figure 4A). As Figure 4B showed, the knockdown of PINK1 or DOR also promoted the release of cytochrome c from mitochondria to cytoplasm (Figure 4B). Compared to the strong inhibitory effects on cytochrome c release mediated by the administration of UFP-512 in the cells transfected with a negative control siRNA, activating DOR in the cells transfected with PINK1 siRNA or DOR siRNA did not induce a major change in the cytosol expression of cytochrome c.

### DOR activation promoted the translocation of Parkin from the cytosol to mitochondria in MPP^+^ condition with an increased phosphorylation at Parkin’s UBL (Ser65) domain, but induced a major loss of Parkin in the cytoplasm of hypoxic cells

**Figure 5:**
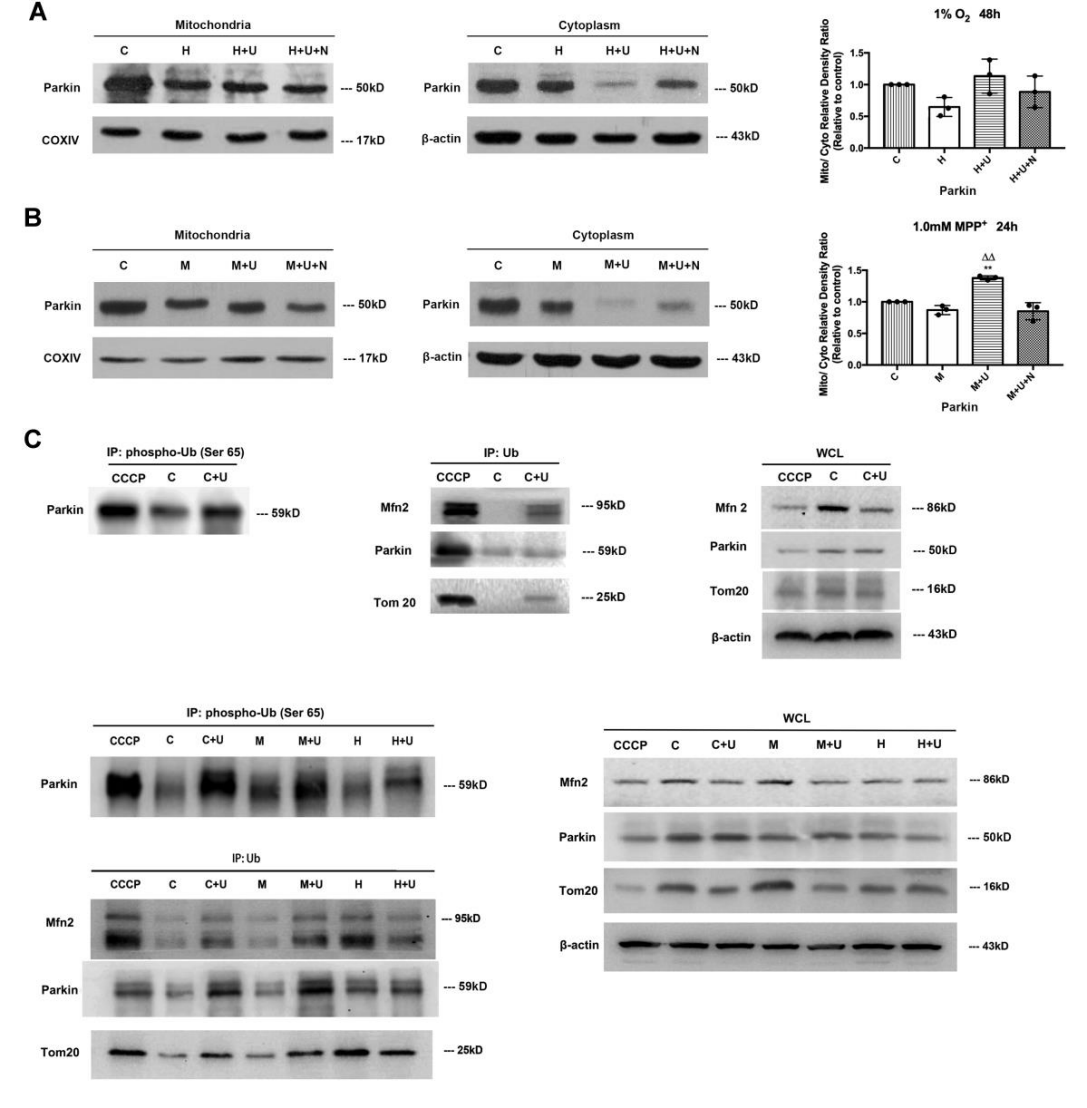
**DOR activation promoted Parkin’s translocation from cytoplasm to mitochondria and its phosphorylation at Ser65 UBL domain and increased OMM ubiquitination for mitophagy.** (**A**) PC12 cells were exposed to hypoxia at 1% O_2_ for 48 hrs, the protein extracted from mitochondria and cytosols were analyzed by Western blot respectively. C: normoxic control. H: hypoxia. H+U: DOR was activated using UFP-512 in hypoxic conditions. H+ U+N: PC12 cells were treated with UFP-512 plus naltrindole at the same time in hypoxic conditions. N=3 for each group. Note that hypoxia at 1% O_2_ for 48 hrs led to a significant decrease in Parkin expression both in the mitochondria and cytosol. Activating DOR using UFP-512 caused a modest increase in the ratio of mitochondria/plasma Parkin density, appearing as a sharp decrease of Parkin in the cytoplasm and an inappreciable increase of Parkin in the mitochondria. (**B**) PC12 cells were exposed to 1.0mM MPP^+^ for 24 hrs. The Parkin expressed both in mitochondria and cytosols were measured using Western blot. C: control. M: MPP^+^. M+U: DOR was activated using UFP-512 and exposed to MPP^+^. M+U+N: PC12 cells were treated with UFP-512 plus naltrindole and exposed to MPP^+^. N=3 in each group. ^**^p<0.01 vs. control. ^ΔΔ^*p*<0.01 vs. M. Note that DOR activation caused a translocation of Parkin from cytoplasm to mitochondria with a significant decrease of Parkin in cytosol and a noticeable increase of Parkin in mitochondria under MPP^+^ insults. (**C**) The PC12 cells were treated with CCCP or exposed to hypoxia at 1% O_2_ for 48 hrs or 1.0 mM MPP^+^ for 24 hrs and the control group were established. The proteins were immunoprecipitated with anti-Ub antibody or anti-phospho-Ub (Ser65) antibody. Immunoprecipitants (IPs) and whole cell lysates (WCLs) were analyzed for Parkin, Mfn2 and Tom20. CCCP: positive control. PC12 cells were treated with 10 μM CCCP for 24 hrs. C: control. C+U: the cells were treated with UFP-512. H: hypoxia. H+U: DOR activation with UFP-512 in hypoxic condition. M: MPP^+^. M+ U: DOR was activated using UFP-512 and then exposed to MPP+. Note that the administration of UFP-512 promoted the phosphorylation of Parkin at its Ser65 UBL domain, and increased the ubiquitination of Mfn2 and Tom20 both under normal conditions and MPP^+^ insults. Hypoxia induced a remarkable degradation in Mfn2 and Tom20 expression with an increase in the ubiquitination of these two proteins. UFP-512 did not appreciably alter the hypoxia-mediated effects.

Accumulating evidence suggests that PINK1 is essential for the initiation of mitophagy in response to mitochondrial damage by recruiting Parkin from the cytosol to the mitochondria, phosphorylation at its UBL (Ser65) domain, and activation of Parkin’s E3 ligase activity for OMM proteins ubiquitination such as Mfn20 and Tom20 [4, 7, 11]. Since we have previously observed that DOR activation significantly up-regulated PINK1 under hypoxic and/or MPP^+^ insults [25], we further investigated if DOR activation altered the Parkin expression and its translocation respectively. In the conditions of 1% O_2_ of hypoxia for 48 hrs, Parkin protein was significantly reduced by hypoxic insults with a further reduction after the application of DOR agonist UFP-512 (Supplementary Figure 4). In such conditions, the Parkin expressed in the cytosol and mitochondria also showed a consistent decrease (Figure 5A). Incubation of DOR agonist UFP-512 further decreased the Parkin in the cytoplasm. However, Parkin was not increased at all in the mitochondria (Figure 5A).

MPP^+^ insults also led to a remarkable decrease in total Parkin protein (Supplementary Figure 4). DOR activation did not influence the overall expression level of Parkin (Supplementary Figure 4). Interestingly, activating DOR caused a further reduction of Parkin in the cytoplasm (-22.4% vs. “M” of the cytosol; *P*<0.05) with a significant increase of Parkin in the mitochondria (+22.7% vs. “M” of the mitochondria; *P*<0.05) (Figure 5B), suggesting a translocation of Parkin from cytosol to mitochondria.

Moreover, we treated the cells as indicated in Figure 5C, the proteins were extracted and immunoprecipitated with anti-phopho-Ub antibody or anti-Ub antibody. Immunoprecipitants (IPs) were subjected to Western blot for phospho-Ub (Ser 65) Parkin, auto-ub Parkin, ub-Mfn2 and ub-Tom20 analysis. We observed that the presence of DOR agonist UFP-512 significantly increased the phosphorylation of Parkin’s UBL (Ser65) domain, promoted the auto-ubiquitination of Parkin and the polyubiquitination of Parkin’s substrates Mfn2 and Tom20 in the normal conditions and MPP^+^ insults (Figure 5C). In contrast, the cells exposed to 1% O_2_ for 48 hrs induced a mild increase in the ubiquitinations of OMM proteins, and the application of UFP-512 did not significantly alter the modification level of Parkin and OMM proteins (Figure 5C).

### DOR activation significantly enhanced mitophagy in MPP^+^, but not hypoxia

**Figure 6:**
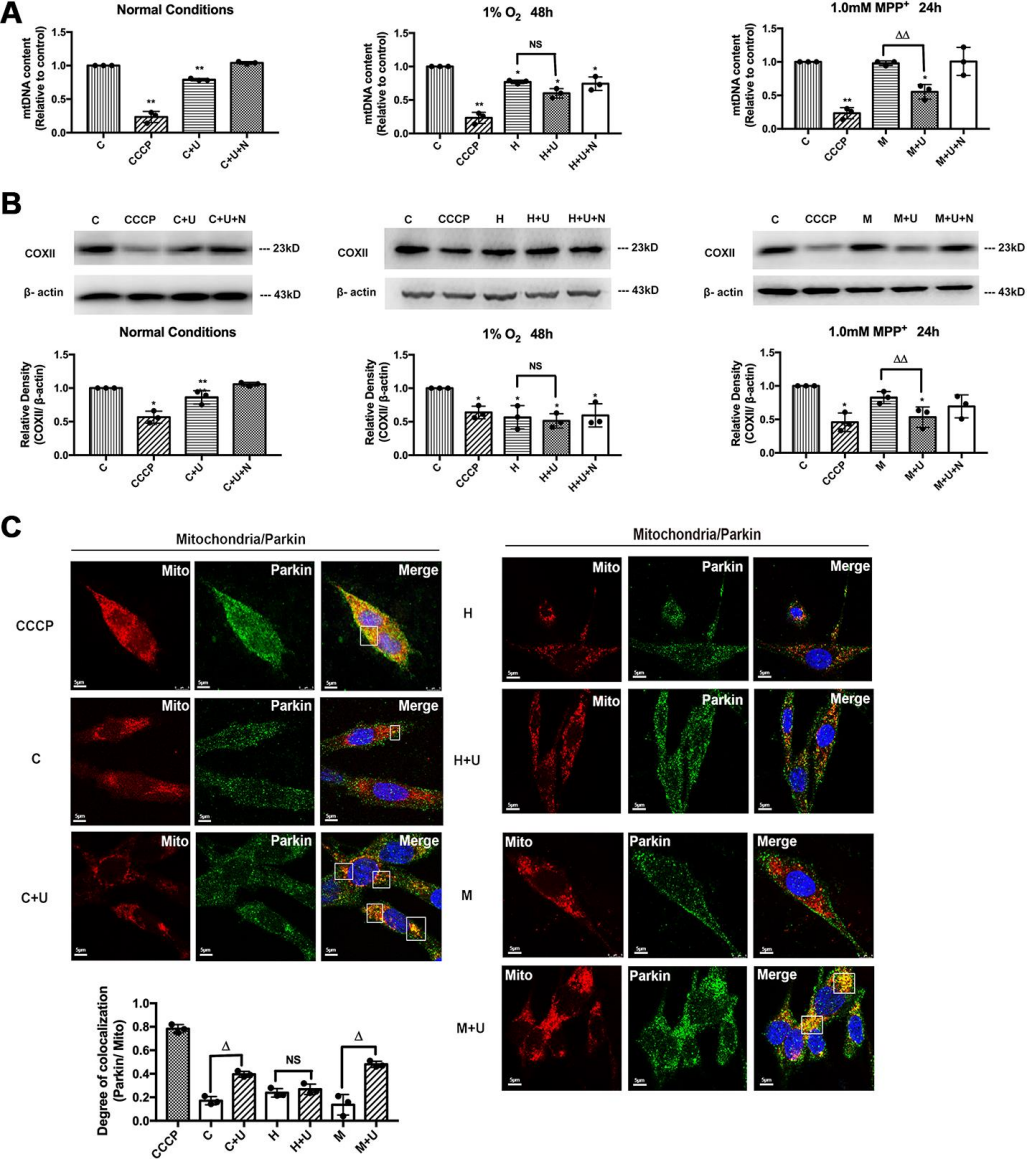
**DOR activation enhanced mitophagy under normoxic and MPP^+^ conditions, but not in hypoxia.** C: control. CCCP: PC12 cells were treated with 10 μM CCCP for 24 hrs. C+U: cells were treated with DOR agonist UFP-512 in normal conditions. C+U+N: cells were treated with DOR agonist UFP-512 plus DOR antagonist naltrindole under normal conditions. H: hypoxia. H+U: DOR was activated using UFP-512 in hypoxic conditions. H+U+N: DOR was treated with UFP-512 plus naltrindole at the same time in hypoxic conditions. M: MPP+. M+U: DOR was activated using UFP-512 and exposed to MPP^+^. M+U+N: PC12 cells were treated with UFP-512 plus naltrindole and exposed to MPP^+^. (**A**) Quantification of mtDNA was carried out using qPCR under normoxic, hypoxic and MPP^+^ conditions. N=3 for each group. NS: not significant, ^*^p<0.05, ^**^p<0.01 vs. control. ^ΔΔ^*p<0.01* vs. M. Note that exposure to CCCP or hypoxia induced significant reduction of mtDNA content in PC12 cell line. The administration of DOR agonist UFP-512 remarkably decreased the mtDNA relative content in the conditions of normoxic and MPP^+^, whereas no appreciable change was observed under hypoxic conditions. (**B**) N=3 for each group. NS: not significant, ^*^p<0.05, ^**^p<0.01 vs. control. ^ΔΔ^*p<0.01* vs. M. Mitophagy was analyzed by measuring the degradation of COXII. Note that CCCP or hypoxic exposure significantly decreased COXII expression. DOR activation by UFP-512 promoted the degradation of COXII under normoxic and MPP^+^ conditions, whereas showed a minimum effect under hypoxia. (**C**) N=3 for each group. NS: not significant. ^Δ^*p<0.05* vs. C or H or M. PC12 cells fluorescent imaging and quantification of co-localization of Parkin/mitochondria were performed in PC12 cell line. Note that exposure to CCCP caused a remarkable increase in co-localization of GRP-Parkin and RFP-mitochondria. The administration of UFP-512 significantly increased the overlap of Parkin/mitochondria under normorxia and MPP^+^, while showed a minimum effect under hypoxia.

Although the concept of DOR-mediated neuroprotection has been well known [33–37], the relationship between DOR signaling and mitophagy was untouched in the previous research. Our recent data [25] and present study well demonstrated that DOR activation led to a series of changes under MPP^+^ insults, including the up-regulation of PINK1, PINK1-induced translocation and modification of Parkin, and the follow-up OMM protein ubiquitination, which constituted a feedforward mechanism during induction of mitophagy. To further examined the possible regulatory effects of DOR on mitophagy, as the first step, we evaluated the ratio change of mtDNA/nDNA, an indicator of mitophagy [38, 39]. We found that the administration of UFP-512 significantly reduced the amount of mtDNA under normal and MPP^+^ conditions, suggesting an enhanced mitophagy induced by DOR activation. Unlike the situation under normoxia or MPP^+^, hypoxia itself induced a significant decrease in mtDNA/ nDNA ratio, whereas the treatment with DOR agonist UFP-512 showed an undetectable effect on the amount of mtDNA (Figure 6A). Moreover, we evaluated the induction of mitophagy in PC12 cells under normal, hypoxic and MPP^+^ conditions by measuring the degradation of cytochrome C oxidase subunit II (COXII), a mitochondrial DNA-encoded inner membrane protein [38] and two OMM protein, Mfn2 and Tom20 in the whole cell lysate (WCL). Consistent with the data of mtDNA/nDNA, after CCCP treatment or DOR activation, COXII, Mfn2 and Tom20, showed a significant decrease in the WCL from the cells exposed to normoxia or MPP^+^ (Figure 6B and Figure 5C), suggesting that the elimination of mitochondria occurred. Hypoxia also led to a degradation of mitochondrial membrane proteins, DOR activation did not further degraded these three mitochondrial membrane proteins under hypoxic conditions (Figure 6B and Supplementary Figure 5C).

As an indicator of PINK1-Parkin mediated mitophagy, the co-localization of GFP-Parkin and RFP-Mito was also quantified in our work. The cells exposed to CCCP showed a marked increase in co-localization of Parkin/mitochondria, while the cells in controlled group and the cells exposed to 1.0mM MPP^+^ showed minimal overlapping (Figure 6C). DOR activation using UFP-512 significantly increased the co-localization of Parkin/mitochondria in the cells exposed to MPP^+^ or normoxia (Figure 6C), suggesting DOR activation enhanced the mitophagy via PINK1-Parkin pathway. Hypoxia induced a significant degradation of COXII and a remarkable decrease in mtDNA, though there was no appreciable overlapping of Parkin/mitochondria in the cells. The application of DOR agonist slightly increased the co-localization of Parkin/mitochondria under hypoxic conditions, though the change was not statistically significant (Figure 6C).

### DOR activation enhanced mitophagy through DOR-PINK1-Parkin pathway

**Figure 7:**
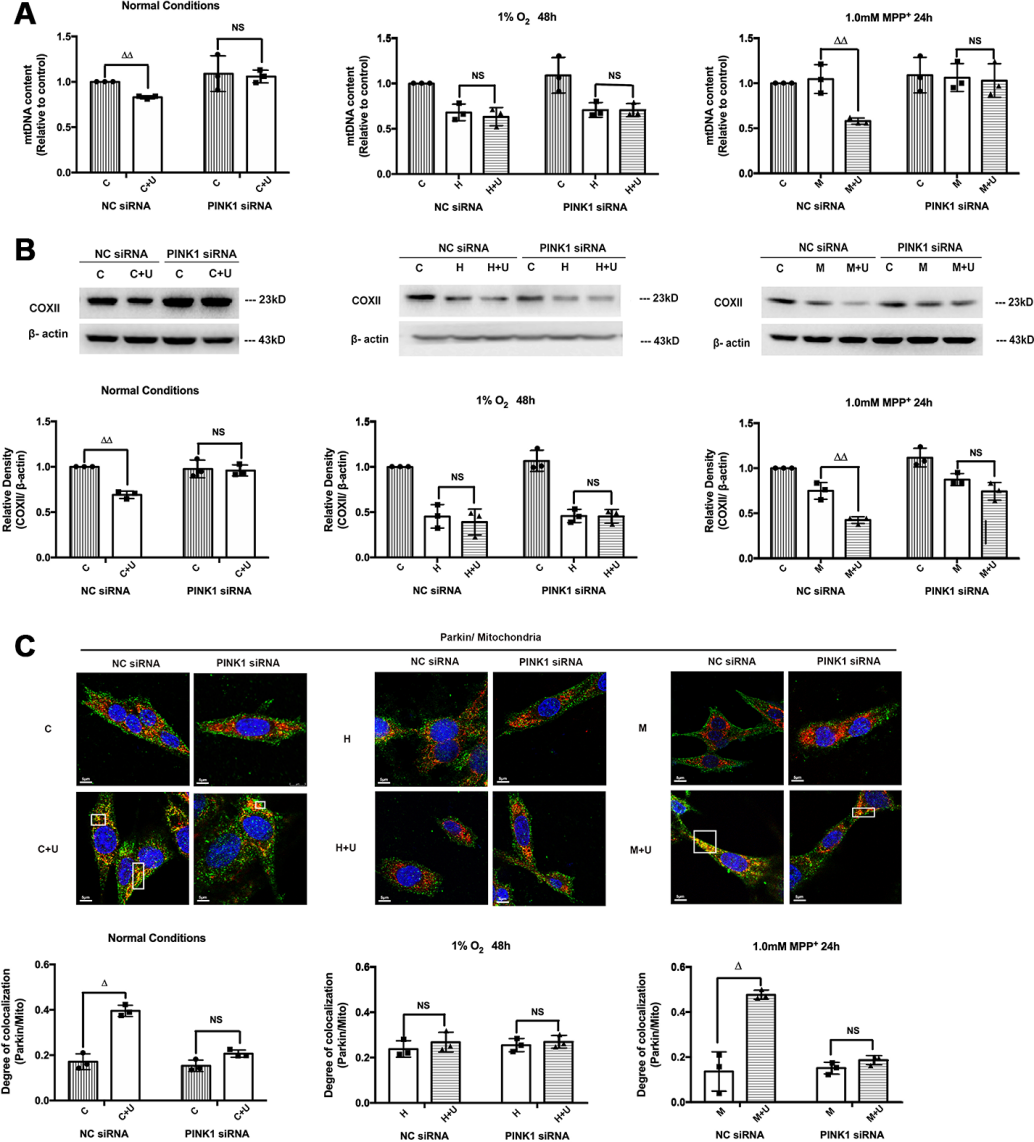
**DOR activation promoted mitophagy under normal conditions and MPP^+^ insults in a PINK1-dependent manner.** C: control. C+U: cells were treated with DOR agonist UFP-512 in normal conditions. H: hypoxia. H + U: DOR was activated using UFP-512 in hypoxic conditions. M: MPP^+^. M + U: DOR was activated using UFP-512 and exposed to MPP^+^. (**A**) PC12 cells transfected with PINK1 siRNA were exposed to hypoxia at 1% O_2_ for 48 hrs or 1.0mM MPP^+^ for 24 hrs, and then the mtDNA content were measured using qPCR. N=3 in each group. NS: not significant, ^ΔΔ^*p<0.01* vs. C or M within the same group. Note that DOR activation induced down-regulation of mtDNA content was significantly attenuated by PINK1 knockdown both under normoxic and/or MPP^+^ conditions. The application of DOR agonist UFP-512 showed an unappreciable effect on mtDNA content after cells were transfected with PINK1 siRNA under hypoxic condition. (**B**) COXII degradation was evaluated before and after PINK1 knockdown. N=3 in each group. NS: not significant. *p<0.01* vs. C or M within the same group. Note that the administration of DOR agonist UFP-512 down-regulated COXII expression under normoxia and MPP^+^, whereas PINK1 knockdown interfered with DOR mediated COXII degradation. (**C**) N=3 in each group. NS: not significant, ^Δ^*p<0.05* vs. C or M within the same group. PC12 cells were transfected with negative control siRNA or PINK1 siRNA. Fluorescent imaging and quantification of co-localization of Parkin/mitochondria were performed in PC12 cell line. Note that PINK1 knockdown seriously interfered with the co-localization of GRP-Parkin and RFP-mitochondria induced by DOR activation under normal and MPP^+^ conditions. The overlapping of Parkin/mitochondria showed no appreciable difference between the cells transfected with NC siRNA and the cells transfected with PINK1 siRNA under hypoxic condition.

We further evaluated the role of DOR in the regulation of PINK1-Parkin mediated mitophagy to ascertain our findings. The cells transfected with DOR siRNA or PINK1 siRNA were subjected to normoxic, hypoxic and MPP^+^ conditions. The results showed that the DOR-induced mitophagy was genetically disrupted by PINK1 knockdown and DOR knockdown, appearing as a rise of the mtDNA content, an increase of COXII expression and a decrease in the overlapping of Parkin/mitochondria under normoxic and MPP^+^ conditions (Figure 7, Supplementary Figure 5). As Supplementary Figure 5B suggested, DOR activation attenuated the down-regulation of PINK1 induced by MPP^+^ and hypoxic insults, while DOR knockdown significantly interfered with the alternations in PINK1 under both conditions. Moreover, although DOR activation effectively enhanced mitophagy under conditions of normoxia and MPP^+^ in the cells transfected with negative control siRNA, these effects were blunted after the transfection with PINK1 siRNA or DOR siRNA (Figure 7A, 7B, and Supplementary Figure 5A, 5B). Consistently, unlike the PINK1 dependent mitophagy under normorxic and MPP^+^ conditions, PINK1 knockdown or DOR knockdown induced a minimum effect on the mitophagy under hypoxia (Figure 7A, 7B, and Supplementary Figure 5A, 5B). Moreover, the knocking down of PINK1 or DOR also disrupted the colocalization of Parkin and mitochondria under normal conditions and MPP^+^ insults, indicating that PINK1 and DOR were critical components of the feedforward mechanisms to induce mitophagy against parkinsonian injury. On the other hand, the overlapping of parkin/mitochondria was not affected at all by PINK1 knockdown or DOR knockdown, suggesting an independent mechanism of mitophagy derived by hypoxia without the participation of PINK1 and DOR (Figure 7C, Supplementary Figure 5C).

## DISCUSSION

In the present study, we made the first finding to show that DOR activation attenuated the MPP^+^- and hypoxia-induced mitochondrial membrane depolarization and improved the mitochondrial function by recovering the ATP generation in the PC12 cell line with a much stronger effect on the cells exposed to MPP^+^ insults. The beneficial effects mediated by DOR on mitochondria were also associated with a differential regulation of ROS under MPP^+^ versus hypoxia. Only in MPP^+^ stress but not in hypoxia, DOR activation specifically eliminated mitochondrial superoxide, which may form the basis for DOR’s more powerful mitoprotective and stronger anti-apoptosis capacity against MPP^+^ than hypoxia. Moreover, we found that DOR activation promoted the translocation of Parkin from the cytosol to mitochondria and increased the phosphorylation of Parkin at the UBL (pSer65) domain, thus enhancing mitophagy in both normoxic and MPP^+^ conditions, but not in hypoxia. The DOR-mediated mitophagy under normal or MPP^+^ conditions were partially blocked by DOR knockdown or PINK1 knockdown, further suggesting the involvement of DOR-PINK1 axis in the regulation of mitophagy under parkinsonian injury.

DOR is one of the three major opioid receptors and was traditionally thought to contribute to pain modulation as well as drug addiction [23, 40]. Our serial studies, however, have well identified DOR as a neural protector that plays an important role in neuroprotection against various stresses [24, 33, 34, 41–43], and these findings has been broadly confirmed by peer researchers worldwide [23, 44–46]. In establishing the novel function of DOR in the regulation of mitochondria, we present the first data of DOR-mediated effects on mitochondrial structure and function. As depicted in the “results” section, although DOR activation significantly attenuated mitochondrial membrane potential loss and recovered mitochondrial ATP production both under hypoxic and MPP^+^ insults (Figure 1, Supplementary Figure 1), the beneficial effects of DOR on mitochondria were more evident under MPP^+^ insults. Moreover, we examined the ROS regulation mediated by DOR in the conditions of hypoxia and MPP^+^, and found that the cells incubated with 1.0 mM MPP^+^ plus 5 μM DOR agonist UFP-512 showed a more significant decrease in mitochondrial superoxide (Figure 2A, 2B and Supplementary Figure 2A, 2B) and a more distinct inhibitory effects on cytochrome c release from mitochondria to cytoplasm (Figure 2C), compared to the cells exposed to 1% O_2_ and treated with 5 μM DOR agonist UFP-512. These data strongly suggest that DOR signaling has a different performance in response to hypoxia and MPP^+^ insults with a stronger protection against MPP^+^-induced parkinsonian injury by targeting mitochondria.

To further explore what makes the differences in mitoprotection by DOR activation against MPP^+^ vs. hypoxic insults, we then attempted to find out the potential linkage among DOR, PINK1, Parkin and mitophagy. PINK1-Parkin pathway contributes greatly to the mitochondrial integrity against multiple stresses such as oxidative stress and neurotoxin [4, 47, 48]. Previously, we have well demonstrated that the DOR-PINK1 axis act as a critical component of the neuroprotection underlying hypoxic and MPP^+^ insults [25]. Associated with these findings, the present work showed that the knockdown of PINK1 or DOR also caused a catastrophic damage to the mitochondria with aggravated mitochondria-based injury under hypoxic and/or MPP^+^ conditions. These damaging effects could not be attenuated or abolished by DOR activation (Figures 3 and 4). The novel observations provided us two hints: (1) The fact that knocking down DOR greatly interfered with UFP-512 effects on mitochondria confirms that DOR agonist UFP-512 specifically activated DOR and ruled out the possible off-target effects; and (2) the observation that PINK1 knockdown also greatly abolished the effects induced by DOR activation on mitochondria suggests that PINK1 might act as a critical down-stream target of DOR in DOR-mediated mitochondrial regulatory pathway.

Furthermore, we evaluated the Parkin protein expressed in mitochondria and cytosol and its phosphorylation at the UBL domain respectively before and after DOR agonist treatment. The results showed that DOR activation promoted the translocation of Parkin from mitochondria to the cytosol and greatly increased the phosphorylation of Parkin’s UBL domain under both normal and MPP^+^ conditions (Figure 5). Briefly, the whole process of PINK1-Parkin dependent mitophagy consist of 3 key steps. Once mitochondrial damage or even mitochondrial membrane potential depolarization occurs, PINK1 perceives the changes and gathered on the surface of dysfunctional mitochondria [4, 6, 7]. Its kinase activity recruits Parkin to mitochondria and phosphorylates its UBL domain and activates E3 Ubiquitin-ligase, thus ubiquitinates downstream OMM including Mfn20 and Tom20, leading to the robust onset of mitophagy [4, 48]. Intriguingly, although DOR activation induced a similar regulation of PINK1 in both MPP^+^ and hypoxic models, DOR activation promoted mitophagy only under MPP^+^, but not under hypoxia. Our evidence suggested that unlike a major increase of Parkin in the mitochondria in response to DOR activation in the cells exposed to MPP^+^ neurotoxin, the increase in mitochondrial Parkin was negligible in the hypoxic cells. This difference may account for the basis of the differential regulation of mitophagy in the PC12 cells by DOR activation.

Another important finding concerning the DOR-mediated mitochondrial regulation is that DOR activation induced a significant co-localization of Parkin/mitochondria with a degradation of mitochondrial inner and outer membrane proteins and mtDNA under the conditions of normoxia and MPP^+^, but not in hypoxia (Figure 6). Together with the regulatory effects of DOR on PINK1 and Parkin, our results indicated an enhancement of mitophagy via DOR-PINK1-Parkin pathway under MPP^+^ insults. Apparently, DOR-PINK1-Parkin dependent mitophagy may increase the cell’s anti-apoptosis capacity by cleaning the damaged mitochondria and thus play a critical role in mitoQC against parkinsonian injury. It is interesting to highlight why this mechanism does not exist in hypoxic condition. We have three potential reasons to explain it. First, DOR activation differently regulated ROS under hypoxic and MPP^+^ insults, which may account for the different level of oxidative stress the mitochondria face, thus leading to different fates of the mitochondria. Secondly, although DOR up-regulated PINK1 in both MPP^+^ and hypoxia conditions, DOR activation surprisingly decreased the total Parkin level and thus failed to accumulate enough Parkin to mitochondria to initiate PINK1-Parkin dependent mitophagy in hypoxic conditions and maintaining mitochondrial homeostasis. Thirdly, emerging evidence suggests that the mitophagy is induced mainly through the BNIP3 and NIX signaling pathway under hypoxia [49–51]. It has also been reported that hypoxia induced extensive mitochondrial degradation in a FUNDC1-dependent manner [52, 53]. Therefore, the regulation of mitophagy in hypoxic environment, may simply be PINK1-Parkin independent.

Although the etiology of PD remains unknown, it has been well linked to genetic and environmental factors and age-related neurodegeneration. Mitochondria within autophagosomes has been found in the neurons of PD patients [54] and abnormal mitophagy was observed in various PD models, including environmental and genetic forms [55–58]. Moreover, the importance of mitophagy in PD pathogenesis is proved by the prevalence of familial cases associated with deficiencies in several mitochondria- related genes [59]. Since mitophagy plays a critical role in PD pathogenesis, DOR might be a potential target for the treatment of PD since its activation enhances the mitophagic ability to clear the damaged mitochondria as well as other debris, thereby recovering the mitochondrial dynamics and protecting the brain from PD. However, we would like to indicate a limitation of this study because we used highly differentiated PC12 cells to establish an *in-vitro* PD model. Although these cells are one of the most commonly used neuron-like cell lines and widely adopted to establish in vitro PD injury, their responses may not be equal to those of “true” neurons in all aspects. Therefore, it is needed to conduct more investigations using real neurons and *in vivo* models to further illustrate the DOR-mediated mitoprotection against parkinsonian injury in future.

**Figure 8:**
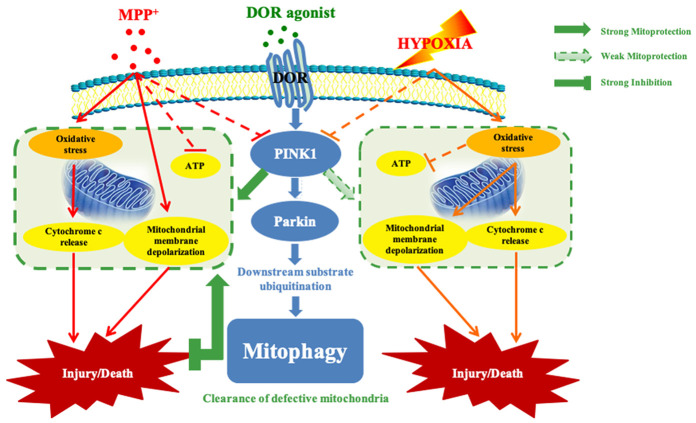
**Schematic mechanisms underlying DOR mediated protection against mitochondrial injury under hypoxic and/or MPP^+^ insults.** Solid green arrow: strong mitoprotection. Dotted green arrow: weak mitoprotection. Green flat arrow: strong inhibition. Note that although DOR activation up-regulated PINK1 in both conditions of MPP^+^ and hypoxia, DOR exhibited a more powerful mitoprotective capacity against MPP^+^ insults through stabilizing mitochondrial potential, restoring mitochondrial function, and inhibiting anti-cytochrome c release from mitochondria to cytosol. Moreover, DOR activation differentially regulated ROS under MPP^+^ vs. hypoxia, and it also specifically promoted the recruitment of Parkin in the mitochondria, thus enhanced the mitophagy in a PINK1-Parkin dependent manner in MPP^+^ conditions but not in hypoxia.

In conclusion, as shown in Figure 8, our study demonstrates that DOR activation attenuates MPP^+^-induced mitochondrial dysfunction by inducing PINK1-Parkin dependent mitophagy. Our study suggests that DOR activation is a potential therapeutic avenue to improve mitochondrial function and prevent neurotoxicity in Parkinson’s disease.

## MATERIALS AND METHODS

### Cell culture

The rat PC-12 cell line was purchased from the Type Culture Collection of the Chinese Academy of Sciences, Shanghai, China and cultured and treated them as described previously [25]. Cells were grown in Dulbecco’s Modified Eagle Medium (DMEM) with 10% FBS. The differentiated cells were maintained in 6-well plates or 12-well plates and randomly allocated to normoxic, MPP^+^ and hypoxic groups. The cells in normoxic group were incubated at 37°C in a humidified incubator with 5% CO_2_. To induce hypoxia, cells were transferred into a hypoxic chamber (Galaxy 48R, New Brunswick, Edison, NJ, USA) with the O_2_ levels being kept strictly at 1% for 48hrs. To induce MPP^+^ injury, cells were exposed to 1.0mM of MPP^+^ for 24 hrs after cell passage. MPP^+^ or 1-methyl-4-phenylpyridinium was purchased from Sigma Chemical Co (Cat: 15H467, St. Louis, MS, USA) and used at 1μM concentration. UFP-512 is a highly specific and potent DOR agonist that was synthesized by our research group [24, 25, 30, 42, 60] and was used to treat PC12 cells at 5μM concentration. The DOR antagonist, Naltrindole hydrochloride, was obtained from Tocris Bioscience (Cat: 0740, Bristol, UK) and used to treat PC12 cells at 5μM concentration.

### Mitochondrial membrane potential measurements

The mitochondrial membrane potential (MMP) of cells was measured by staining with a cell-permeable, MMP-sensitive dye, Tetramethylrhodamine methyl ester (TMRM; Cat.No. I34361; Thermo Fisher Scientific, Waltham, MA, USA). The cells were incubated with 1× TMRM (prepared from diluting 1000X TMRM stock solution) for 30 mins at 37°C. Then, after washing the cells thrice with 1× PBS, images were captured with a fluorescence microscope (Olympus IX71, Japan) using the TRITC/RFP filter settings. The TMRM-stained cells were also analyzed using the 488nm excitation laser with a flow cytometer (BioTek, Winooski, VT, USA).

### Cellular ATP measurements

The cellular ATP concentration was quantified using the ATP assay kit (Cat: S0026; Beyotime Co., Shanghai, China) according to the manufacturer’s instructions. Briefly, the cells per well in the 6-well plate were incubated with the lysis buffer provided in the kit. Then, the cell lysate was centrifuged at 12,000 xg for 5 mins at 4°C and the supernatant was collected for the measurements. We incubated 10μl of the supernatant with 100μl of the ATP assay working solution in a 96-well cell-culture plate at room temperature. Then, the relative light unit (RLU) of the standards and the samples was measured using a luminometer (BioTek, Winooski, VT, USA). The ATP concentration of the samples was calculated using a standard curve.

### Estimation of mitochondrial superoxide

We used the mitochondrial superoxide-sensitive dye, MitoSox Red (M36008; Thermo Fisher Scientific, Waltham, MA, USA), to measure superoxide levels in the mitochondria. The cells were incubated with 1ml of 5μM MitoSOX Red for 10 minutes at 37 °C in the dark. Then, the cells were gently washed thrice with 1X PBS and imaged using a fluorescence microscope (Olympus IX71, Japan) or analyzed by flow cytometry using the flow cytometer (BioTek, Winooski, VT, USA).

### Western blotting

Total cellular protein was extracted using the RIPA lysis buffer containing 0.5% 100mM PMSF, 0.1% protease inhibitor, and 1% phosphatase inhibitor (KeyGEN Biotec, Cat: KGP2100, Nanjing, China). The protein concentration was determined using the BCA protein assay kit (Cat:1859078, Thermo Scientific) according to the manufacturer’s protocol. No-primary control was established every time to verify signal bands when using a new antibody. Equal amounts of protein samples in 5x SDS-PAGE loading buffer were separated on a 10%-12.5% SDS-PAGE and transferred onto polyvinylidenedifluoride (PVDF) membranes. The membranes were then blocked with 5% skimmed milk in 1X TBST for 1 h. Then, the membranes were incubated overnight at 4^o^C with the following primary antibodies: anti-COXII (Cat:55070-1-AP, Proteintech, Rosemont, IL, USA), anti-Ub (Cat:sc-8017, Santa Cruz, Dallas, Texas, USA), anti-Mfn2 (Cat:sc-100560; Santa Cruz), anti-Tom20 (Cat:sc-17764; Santa Cruz), anti-PINK1 Novus Biologicals (Cat: BC100-494SS; Novus Biologicals, Centennial, CO, USA). anti-Parkin (Cat: 4211, Cell Signaling Technology, Danvers, CO, USA) anti-COX IV (Cat: 4844, Cell Signaling Technology), anti-cytochrome C (Cat: 4272; Cell Signaling Technology), anti-DOR (Cat: AB1560; EMD Millipore Corporation, Temecula, CA, USA), and anti-phospho-Ser65-ubiquitin (Cat: ABS1513, Temecula, CA, USA). Then, the membranes were incubated with HRP-conjugated secondary antibodies (Cat: 611545215, Jackson ImmunoResearch, PA, USA) at room temperature for 1 h. The blots were then developed using the Western Lightening® Chemiluminescence Reagent Plus (Perkin-Elmer, Boston, MA, USA) and the protein bands were quantified using the Image J software (NIH, USA).

### Cell transfections

The control siRNAs (si-NC) and siRNAs against PINK1 (si-PINK1) and DOR (si-DOR) were designed as previously published [25] and purchased from Genepharma Co. Ltd. (Shanghai, China). The PC12 cells were transfected with the control (si-NC), si-PINK1, or si-DOR constructs using Lipofectamine (Genepharma Co, Shanghai, China) according to manufacturer’s instructions. The siRNA compounds were diluted in Opti-MEM and then mixed at a 1:1 ratio with diluted Lipofectamine in Opti-MEM. The mixture was added directly to the culture medium for 20 minutes stabilization. After 6-hr incubation, the medium containing siRNA was removed and the fresh medium was added. Successfully transfected PC12 cells were used for the follow-up experiments.

### Cell fractionation

We isolated cytosolic and mitochondrial fractions using the mitochondria isolation kit (C3601, Beyotime Co., Shanghai, China). Briefly, the cells were first rinsed with pre-cooled PBS and the cell pellet was resuspended in the mitochondrial isolation reagent. Then the mixture was homogenized on ice in a Dounce mixer grinder. Then, the cell suspension was centrifuged at 600 xg for 10 mins at 4°C. The supernatant was further transferred into another tube and centrifuged at 11000 xg for 10 mins at 4°C. The pellet was harvested as the mitochondrial fraction, resuspended in the lysis buffer, and stored at -80^o^C for future use. The remaining supernatant was centrifuged again at 12000 xg for 10 mins at 4°C. The pellet was discarded and the supernatant was used as the cytoplasmic fraction.

### Mitochondrial DNA quantification

The mitochondrial DNA content relative to nuclear DNA was quantified using real time quantitative polymerase chain reaction (q-PCR) as previously described [61, 62] using the following primers: mtDNA-sense: 5' ACTATTCTTCCACCACAACA 3'; mtDNA-antisense: 5' TCCTACTCCTTCTCATCCAA 3'; nDNA-sense: 5' TGGAGATGCTGTGGTGAT 3'; nDNA-antisense: 5' ATACTGAGTGTTACTGTAGGAG 3'; The relative mtDNA content was estimated from the Ct values for the mtDNA and nDNA using the 2^-ΔΔCt^ method as previously described [25].

### Immunofluorescence

The PC12 cells were seeded on microscope cover glass in 12-well plates. After the different treatments, the cells were incubated with 100 nM Mitotracker deep red (Cat: M22426, Invitrogen, UK) for 30 min, and fixed with 10% neutral formalin for 20 mins at room temperature. The cells were then rinsed with PBS for thrice, permeabilized with 0.1% Triton™ X-100 for 15 minutes, blocked with 1% BSA, incubated with 2 μg/ml rabbit-anti-Parkin antibody (14060-1-AP, Proteintech, Rosemont, IL, USA for 1 h at room temperature, and then incubated at 4°C overnights. Then, the cells were incubated with 4 μg/ml goat anti-rabbit IgG (H+L) Highly Cross-Adsorbed Secondary Antibody Alexa Fluor® 488 conjugate (Cat: A11034, Thermo Scientific, Waltham, MA, USA) in 1X PBS containing 0.1% BSA for 1 h at room temperature. Finally, the cells were stained with the DAPI staining solution (Cat: C1005; Beyotime Co. Shanghai, China) for 5 mins. The cells were then visualized and photographed using the Leica TCS-SP2 confocal scanning microscope (Leica, Heidelberg, Germany). For analysis, we selected five randomly selected fields from each sample and calculated the average fluorescence intensity (GFP or RFP) to determine the degree of co-localization.

### Statistical analysis

All the data are presented as means ± SEM. All experiments were performed at least thrice. The data for multiple groups were compared using the one-way ANOVA followed by Bonferroni’s test. All statistical analysis was performed using the GraphPad Prism 5.0 version (GraphPad Software, La Jolla, CA, USA).

## Supplementary Material

Supplementary Figures
