# Supplementary material for: δ-opioid receptor activation protects against Parkinson’s disease-related mitochondrial dysfunction by enhancing PINK1/Parkin-dependent mitophagy
Source: Aging (Albany NY). 2020 Nov 10;12(24):25035–59. doi: 10.18632/aging.103970 (PMC7803568; doi:10.18632/aging.103970)
Supplement: Supplementary Figures [file aging-12-103970-s001.pdf]

SUPPLEMENTARY FIGURES

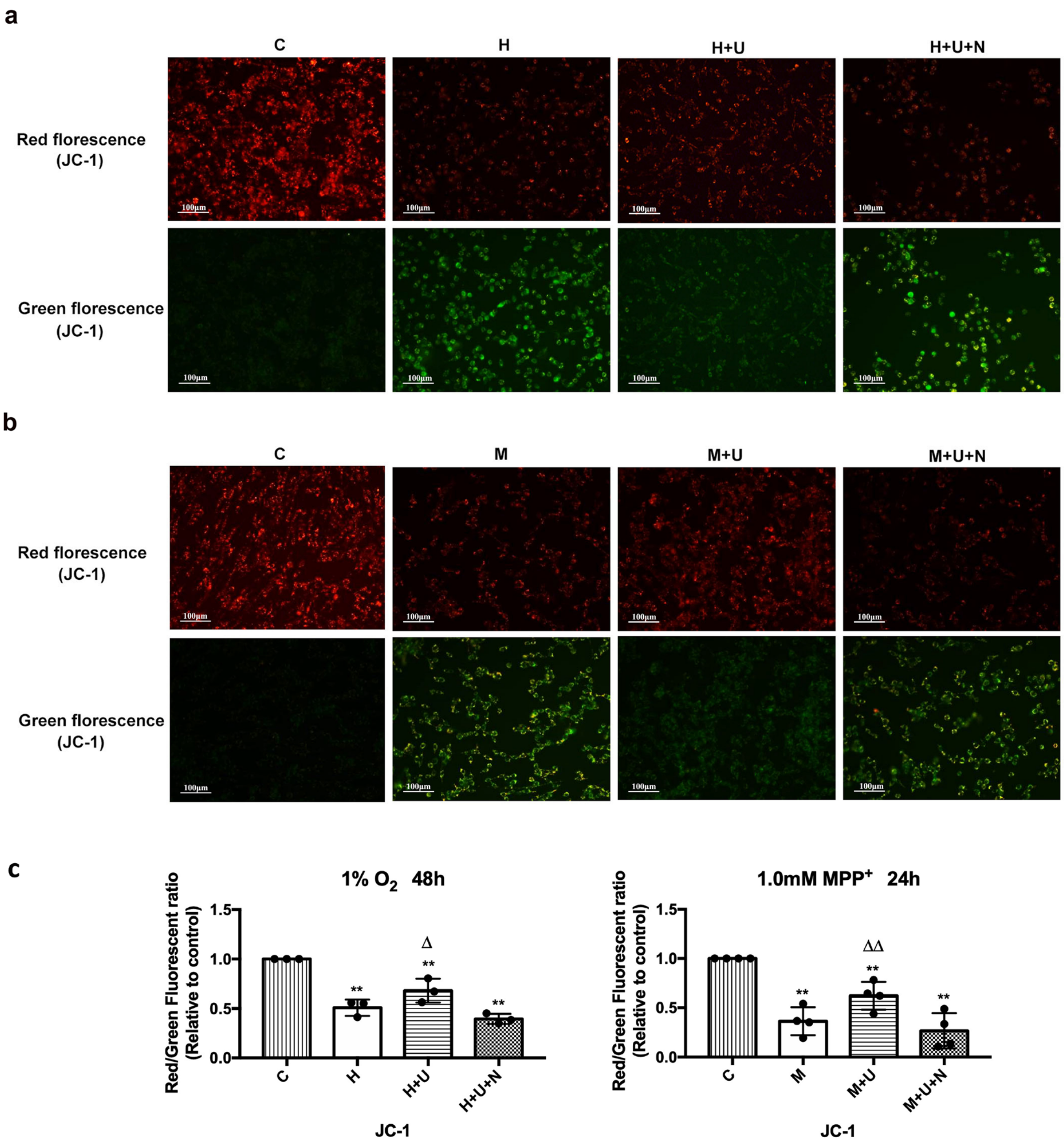

Supplementary Figure 1. DOR activation attenuated hypoxia- and/or MPP⁺-induced mitochondrial membrane potential depolarization. (**A**) PC12 cells were exposed to hypoxia at 1% O_2 for 48 hrs, the mitochondrial membrane potential was measured by JC-1. C: normoxic control. H: hypoxia. H + U: DOR was activated using UFP-512 in hypoxic conditions. H + U+ N: PC12 cells were treated with UFP-512 plus naltrindole at the same time in hypoxic conditions. Note that hypoxia significantly decreased the red fluorescence with a remarkable increase in green fluorescence, the application of DOR agonist UFP-512 attenuated these changes. (**B**) PC12 cells were exposed to 1.0 mM MPP^+ for 24 hrs. C: control. M: MPP^+. M + U: DOR was activated using UFP-512 and exposed to MPP^+. M+U+N: PC12 cells were treated with UFP-512 plus naltrindole and exposed to MPP^+. N=3 in each group. Note that MPP^+ insults also caused a conversion of JC-1 from J-aggregates to monomer, while activating DOR using UFP-512 significantly reversed these changes under MPP^+ insults. (**C**) The ratio of red/green fluorescence was measured by flow cytometer. N=3 in each group. $^{**}p<0.01$ vs. C; $^{\Delta}p<0.05$ vs. H; $^{\Delta\Delta}p<0.01$ vs. M. Note that the ratio of red/green fluorescence reflected alternations in the mitochondrial membrane potential. The results measured by flow cytometer were consistent with the florescence microscope observation. Hypoxia and MPP^+ caused a depolarization of mitochondrial membrane potential, while DOR activation with UFP-512 ameliorated such destructive change.

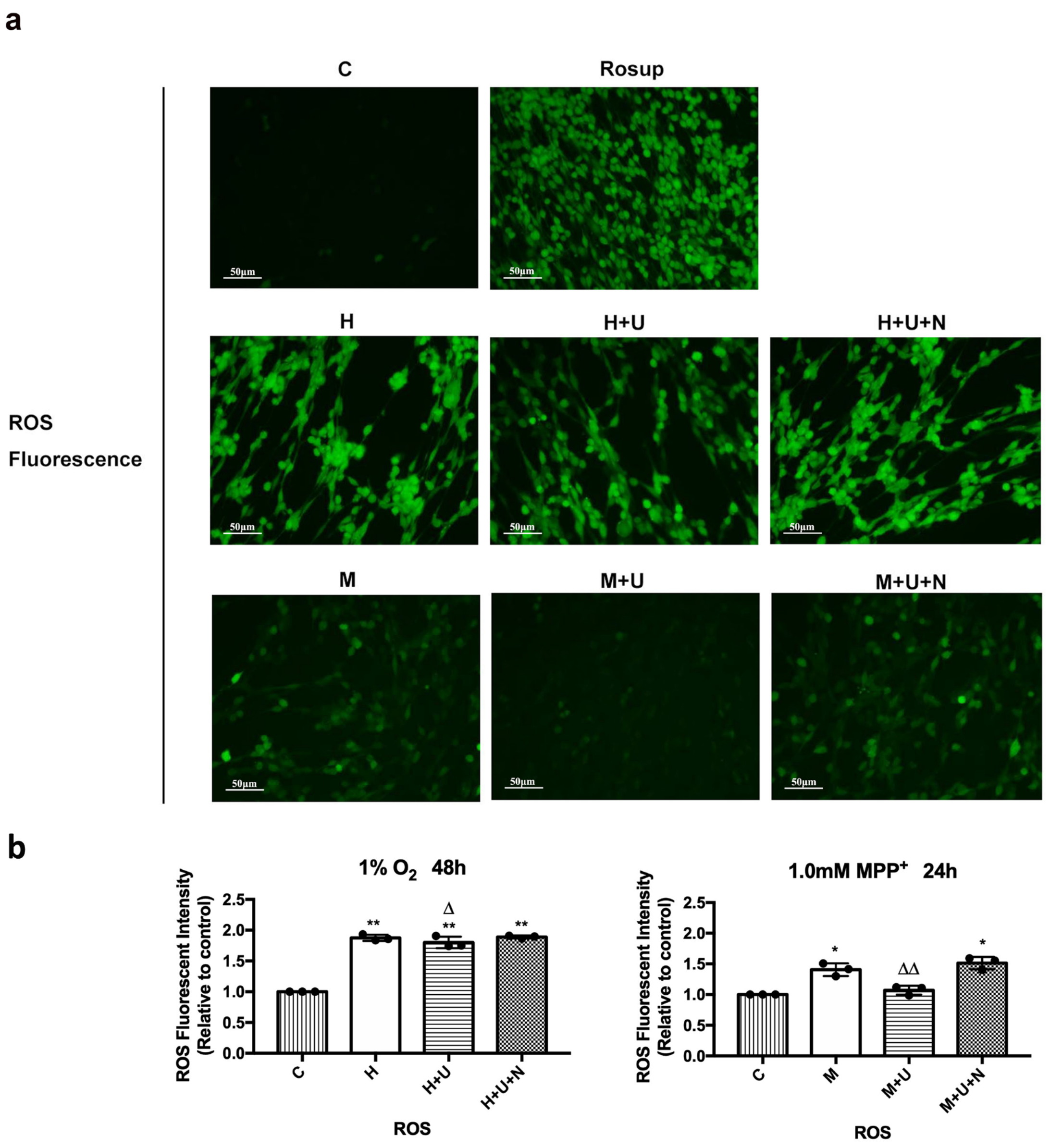

Supplementary Figure 2. DOR activation down-regulated ROS in the cells exposed to hypoxia and/or MPP$^+$. (**A**) PC12 cells were exposed to 1% O_2 for 48 hrs or treated with 1.0mM MPP$^+$ for 24 hrs. C: control. Rosup: the cells were treated with 5μg/ml Rosup for 30 min before measurement as the positive control. H: hypoxia. H + U: DOR was activated using UFP-512 in hypoxic conditions. H + U+ N: PC12 cells were treated with UFP-512 plus naltrindole at the same time in hypoxic conditions. M: MPP$^+$. M + U: DOR was activated using UFP-512 and exposed to MPP$^+$. M+U+N: PC12 cells were treated with UFP-512 plus naltrindole and exposed to MPP$^+$. Note that hypoxia significantly increased the ROS release appearing as a sharp increase in green fluorescent of DCF. DOR activation led to a slight decrease of ROS in PC12 cells. MPP$^+$ also caused an increase of ROS, DOR activation significantly attenuated the ROS release, and the addition of naltrindole plus UFP-512 reversed the effects induced by DOR activation. (**B**) The ROS fluorescent intensity was measured by flow cytometer. N=3 for each group. **p*<0.05 vs. C; ***p*<0.01 vs. C; $^{\Delta}p<0.05$ vs. H; $^{\Delta\Delta}p<0.01$ vs. M. Note that the statistic results of ROS fluorescent intensity were consistent with those of the microscope observation.

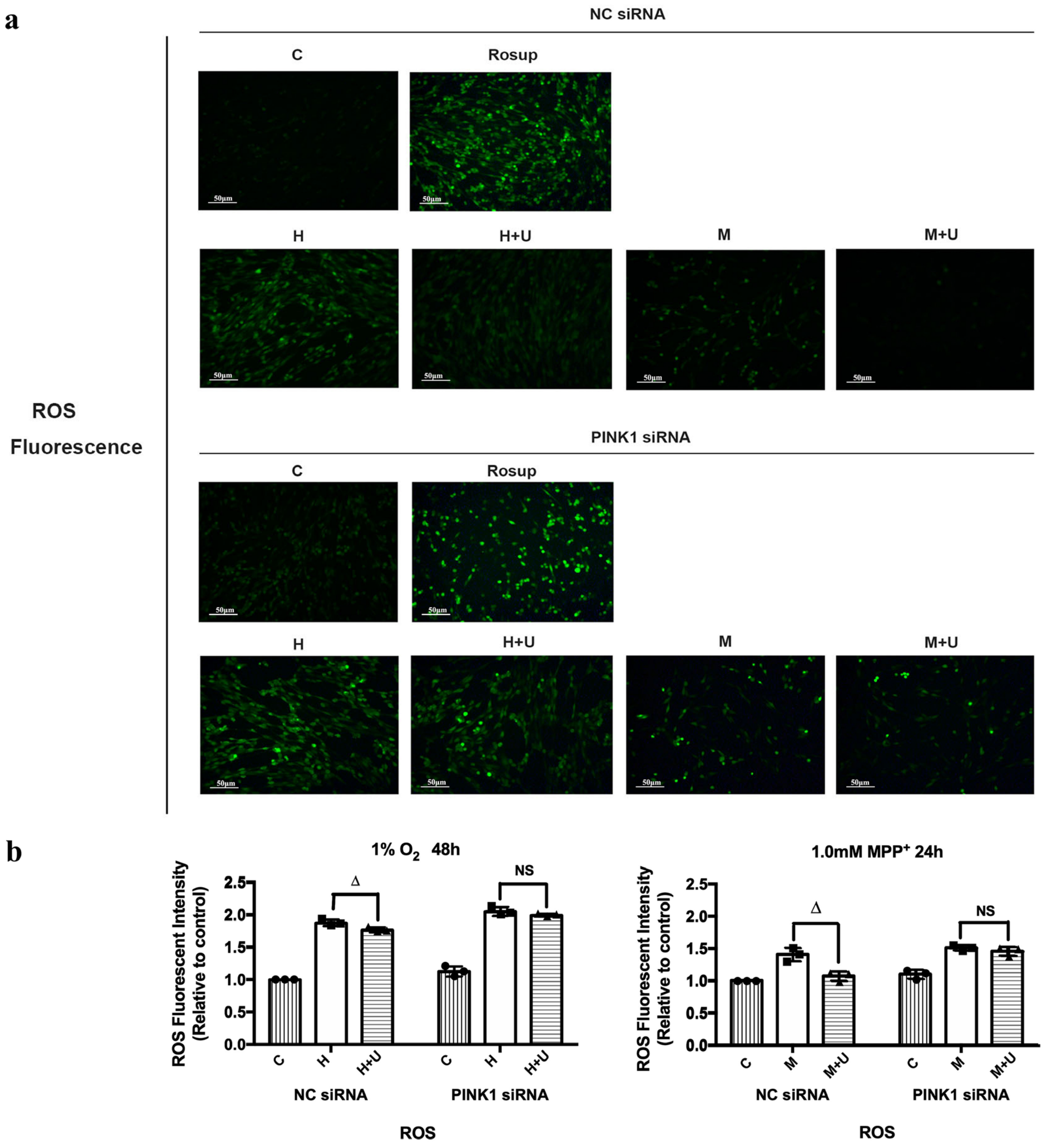

Supplementary Figure 3. Knockdown of PINK1 interfered with DOR-mediated ROS regulation against hypoxia and/or MPP$^+$ insults. (**A**) The PC12 cells transfected with control siRNA or PINK1 siRNA were exposed to hypoxia at 1% O_2 for 48 hrs or 1.0 mM MPP$^+$ for 24 hrs, and then the ROS fluorescence were detected under microscope and the fluorescent intensity was quantitated by flow cytometer. C: control. Rosup: positive control. PC12 cells were treated with 5μg/ml Rosup for 30 min before measurement. H: hypoxia. H + U: DOR activation with UFP-512 in hypoxic condition. M: MPP$^+$. M + U: DOR was activated using UFP-512 and then exposed to MPP$^+$. Note that the ROS fluorescence baseline was significantly enhanced by PINK1 knockdown both under hypoxic and MPP+ conditions. The application of DOR agonist UFP-512 showed an unappreciable effect on ROS release. (**B**) N=3 in each group. NS: not significant, $^{\Delta}p<0.05$ vs. H or M within the same group. Note that the statistical data analyzed by flow cytometer were consistent with the results observed under microscope. PINK1 knockdown greatly aggravated the ROS release and interfered with DOR-mediated down-regulation of ROS.

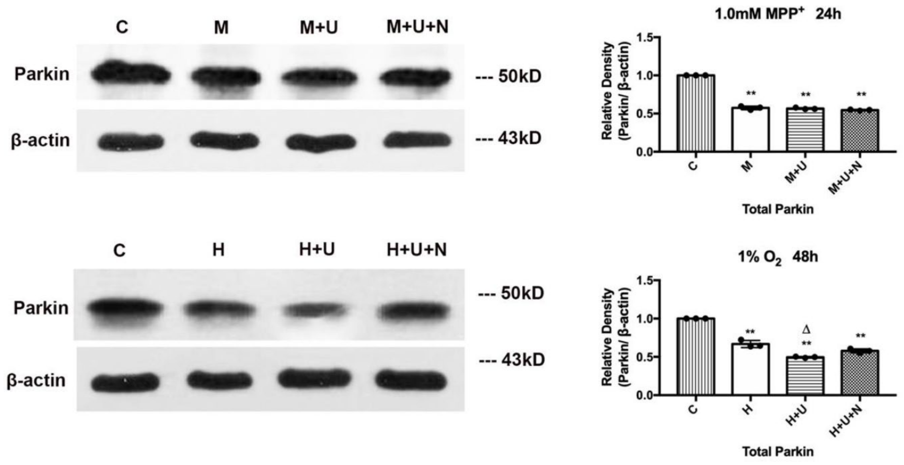

Supplementary Figure 4. The effects of DOR activation on Parkin expression under hypoxia and/or MPP+. The PC12 cells were exposed to hypoxia at 1% O_2 for 48 hrs or 1.0mM MPP^+ for 24 hrs, and then the Parkin expression level was examined by Western blot. C: control. H: hypoxia. H + U: DOR activation with UFP-512 in hypoxic condition. H+U+N: PC12 cells were treated with UFP-512 plus naltrindole at the same time in hypoxic condition. M: MPP^+. M + U: DOR was activated using UFP-512 and then exposed to MPP^+. M+U+N: PC12 cells were treated with UFP-512 plus naltrindole and exposed to MPP^+. N=3 in each group. $^{**}p<0.01$ vs. C, $^{\Delta}p<0.05$ vs. H within the same group. Note that both MPP^+ and hypoxia largely decreased the level of Parkin protein, while DOR activation with UFP-512 did not significantly change the total amount of Parkin under MPP^+ insults, but remarkably decreased total Parkin under hypoxic condition.

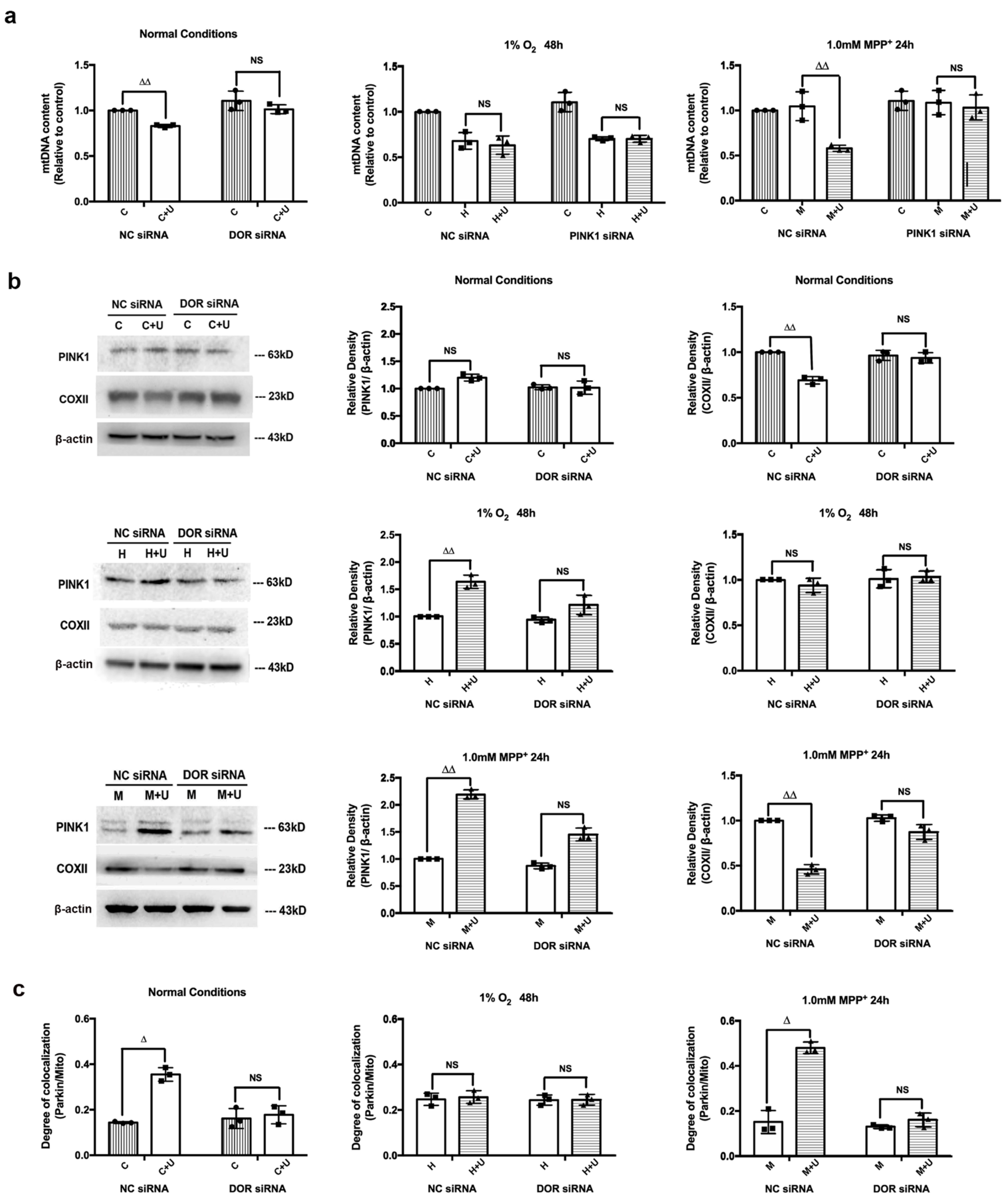

Supplementary Figure 5. DOR knockdown largely interfered with the DOR-mediated mitophagic promotion. C: control. C+U: The cells were treated with DOR agonist UFP-512 in normal conditions. H: hypoxia. H + U: DOR was activated using UFP-512 in hypoxic conditions. M: MPP^+. M + U: DOR was activated using UFP-512 and exposed to MPP^+. (**A**) The PC12 cells transfected with DOR siRNA were exposed to hypoxia at 1% O_2 for 48 hrs or 1.0 mM MPP^+ for 24 hrs, the mtDNA content was measured using qPCR. N=3 in each group. NS: not significant, $^{\Delta\Delta}p<0.01$ vs. C or M within the same group. Note that DOR activation induced down-regulation of mtDNA content was significantly attenuated by DOR knockdown in normoxic and/or MPP^+ conditions. (**B**) PINK1 and COXII density were evaluated before and after DOR knockdown. N=3 in each group. NS: not significant, $^{\Delta\Delta}p<0.01$ vs. C or H or M within the same group. Note that DOR activation with UFP-512 up-regulated PINK1 expression under hypoxic and MPP^+ conditions, while down-regulated COXII expression in normal and MPP^+ conditions. DOR knockdown interfered with DOR activation mediated alternations in PINK1 and COXII. (**C**) N=3 in each group. NS: not significant, $^{\Delta}p<0.05$ vs. C or M within the same group. PC12 cells were transfected with negative control siRNA or DOR siRNA. Fluorescent imaging and quantification of co-localization of Parkin/mitochondria were performed in the PC12 cells. Note that DOR knockdown seriously interfered with the co-localization of GRP-Parkin and RFP-mitochondria induced by DOR activation in normal and MPP^+ conditions.
